# Racial disparities in gun violence: current evidence, an integrated cascading perspective, knowledge gaps, policy prescriptions

**DOI:** 10.3389/fpubh.2026.1957448

**Published:** 2026-09-02

**Authors:** Alex R. Piquero

**Affiliations:** University of Miami, Coral Gables, FL, United States

**Keywords:** firearm violence, gun violence, minorities, non-fatal firearm violence, racial/ethnic minorities

## Abstract

At the intersection of public health and violence is the glaring disparity surrounding racial disparities in gun violence. This mini-review summarizes what is known about racial disparities in gun violence, identifies some of the key theoretical frameworks advanced to explain the disparities and located them in an integrated cascading perspective, identifies several research gaps, and concludes by outlining a series of future research issues.

## Introduction

Issues at the intersection of race and crime are among the most controversial across the full range of academic disciplines and within policy circles (1, 2). Four of the largest issues surround: (1) the school-to-prison pipeline (3), which focuses on school inequalities in discipline and punishment that are exacerbated among Black people which, in turn, increases the likelihood of juvenile punishment and the knifing off of prosocial opportunities and outcomes; (2) the over-representation of minorities – especially Black people – in the criminal justice system, a disparity that has been attributed to differential enforcement by criminal justice personnel and the system more generally against Black people, differential involvement by Black people in the types of (serious) offenses that come to the attention of criminal justice actors, or some combination thereof (see 4); (3) racial profiling, which argues that the police selectively stop, frisk, and arrest racial minorities—especially Black people—at much higher rates than they do for White people (5), and (4) the over-representation of Black people in both fatal and non-fatal firearm injuries and deaths. Yet, despite comprising less than 15% of the US population, Black people account for almost half of gun violence events, a disparity so striking insofar as quite stable over time.

This mini-review takes up the fourth issue noted above, the racial gun violence disparity, and focuses on: (1) key trends in gun violence disparity, (2) different schools of thought or controversies as to why this disparity exists, (3) current research gaps, and (4) potential future developments in the field.

## Research evidence: key trends in gun violence disparity

As noted earlier, social scientists and public health scholars have long observed (and debated) the existence of racial disparities in both offending and victimization, especially with respect to violent crime (6–8). Here, I review four key pieces of research that are focused on racial/ethnic disparities in gun violence.

First, Lanfear et al. (9) used multi-cohort data from the Project on Human Development in Chicago Neighborhoods (PHDCN) in order to examine demographic differences in ‘firearm violence exposure', which included several items such as age when first shot, age when first saw someone shot, and past-year frequency of fatal and non-fatal shootings close to the respondent's residence. Results showed that both Black people and Hispanics evidenced higher odds of firearm violence exposure. Specifically, compared to White people, Black people had higher rates of all three types of firearm exposure, while Hispanics had higher rates for two of the three (but not age when first shot).

Piquero (10) used violent victimization data from the National Crime Victimization Survey as well as firearms homicide data collected via death certificates by the U.S. Center for Disease Control (CDC) for Black people, Hispanics, and White people in order to assess whether disparities in violent crime victimization among minorities are higher or whether there is distinct difference for firearm violence. Although there were very small differences in victimization rates by race/ethnicity for most types of violent crime within the NCVS, Piquero observed a large ratio of firearm homicides, with Black people experiencing a rate 12 times higher than what was observed for White people, while Hispanics were twice as likely to be victimized by firearm homicide than White people.

Within the context of significant increases in violent crime during the COVID-19 pandemic, Piquero and Roman (11) were interested in deciphering whether the increases in violent crime—especially death by firearm violence—differed across race and ethnicity before, during, and subsequent to the COVID-19 pandemic peak. Their results were striking: death by firearm was concentrated among Black individuals aged 15 to 24 years before, during, and subsequent to the COVID-19 pandemic. In particular, the ratio of firearm homicide between Black people and White people aged 15 to 19 years was 27:1. In other words, for every one White firearm homicide death, there were 27 Black firearm homicide deaths. Recently, in a non-peer reviewed Substack post Roman (12) extended the afore-mentioned analysis through 2025, which incorporates both the homicide spike and the ensuing rapid decline. His results showed a general decline in firearm homicide across all groups/ages, and the Black: White ratio dropped from 27:1 to 21.1:1.

Finally, Knorre and MacDonald (13) used 45 years of the CDC data (1979–2023) to examine the disparity in Black-White firearm homicide rates and also to estimate the fraction of excess deaths among Black people that were attributable to firearm homicides. While the Black-White disparities were quite steady between 1990 and 2010 (still favoring Black males at factors of about 8 or 9 times that of White males), the disparity started to increase thereafter and peaked in 2020 where the firearm homicide rate was over 10 times higher among Black (compared to White) males. Further, the authors calculated over 31,000 excess firearm homicides among Black people during the 2021–2023 time period.

## Developing an integrated cascading perspective underlying racial/ethnic disparities in gun violence

There has been no shortage of theoretical explanations advanced to explain why racial and ethnic disparities in gun violence exist, and within criminology these have tended to simply involve applications of existing frameworks, such as social disorganization, routine activities, and conflict theory that tend to prioritize community and structural factors over individual and situational factors.

Importantly, but in isolation, the different schools of thought begin at higher levels of analyses (structural and macro), move onto localized community level factors, consider situational characteristics, and then identify potential individual characteristics (to include developmental, psychological, and biological factors) all of which, when taken as a whole, may serve as a type of causal cascade which seek to provide a generative account of why the disparity takes the racial, gender, and age-graded shape it does. Here, I briefly outline how four specific explanations can be sequenced into one integrated cascading perspective.

This sequenced cascade begins at the structural level. Williams and Mohammed (14) advanced the framework for study of racism and health (FSRH), which broadly identifies structural, institutional, and cultural racism as fundamental contributors to racial health disparities, which helps to produce racialized economic segregation and concentrated disadvantage which, in turn, helps to create the conditions for gun violence disparities.^1^

This subcultural substrate, then, helps generate both street-code cultural adaptations and legal cynicism that can be thought of in two respects. The first is Anderson's (15) “code of the street” perspective, which entails a set of informal rules that govern interpersonal behavior in marginalized inner-city communities. In these communities, violence regulated via the “code of the street” which prioritizes respect on the street that is gained and maintained through the threat and use of violence at all costs. The second is Carlson's (16) illustration of how race, class, and gender dynamics influence gun carrying that underpin the structurally divergent conditions that lead to racial disparities in firearm violence. Her notion of the “citizen-protector” describes an armed masculinity view where protection becomes the motive for gun-carrying and that threats to masculinity lead to favorable gun attitudes (and penultimately gun use).^2^ These cultural adaptations may be especially salient within a larger structural context of racism and community violence as documented by Quam et al.'s (44) semi-structured interviews with adolescent Black males as they make the transition to adult manhood, where responsibility, emotional reaction, and racial injustice are placed front and center.^3^

These attitudes help to erode what Sampson et al. (17) refer to as “collective efficacy,” which prioritizes the intermix of social cohesion among neighbors coupled with their willingness to intervene for the common good, as a construct that helps guard against violence, for which there is ample and promising evidence. All of this, then, becomes diffused throughout the community at the neighborhood level, and no discussion about racial/ethnic disparities in gun violence can omit the importance of neighborhood context, especially because gun violence is highly concentrated in certain neighborhoods and in certain blocks—and mainly in disadvantaged minority communities (18, 19). Here, Fagan and his colleagues (20) were among the first set of researchers to advance a theory of social contagion of violence which models how violent behavior operates through networks and neighborhoods, finding that Black people are disproportionately affected by violence through this contagion mechanism because they disproportionately reside in neighborhoods where extreme poverty and concentrated racial residential segregation inhibits strong social controls. Related research by Braga, Papachristos, and other scholars also illuminated a theoretical and empirical pattern that gun violence is concentrated within networks and that this concentration is related to “social contagion (i.e., the spread of beliefs, attitudes, and behaviors through social interactions)” [(21), p. 327]. Through the advent of cellphone technology, this contagion has made its way onto social media sites which are used to make violent threats and insults that transition from these sites to the streets leading to gun violence—a term that Patton et al. (22) have referred to as “internet banging.”

Finally, all of this chronic exposure becomes embedded in adolescent development and continues throughout the life-course (23), shaping the individual-level decisions that occur on a daily basis across situational contexts (24–27).

**Figure 1:**
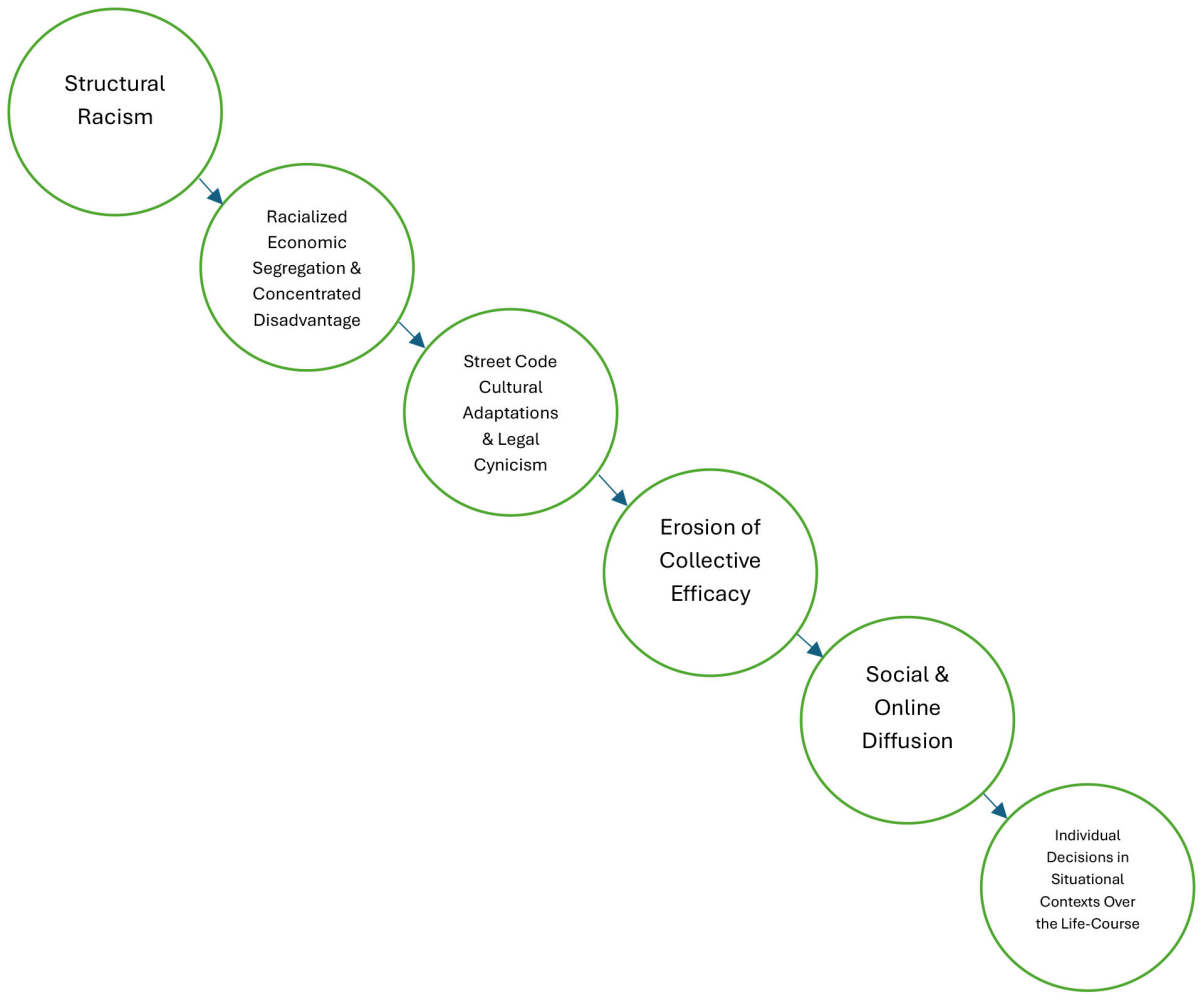
An integrated cascading perspective underlying racial/ethnic disparities in gun violence.

Figure 1 outlines this integrating cascading perspective.

## Current research gaps

Generally speaking, researchers have worked diligently to understand the causes and correlates associated with gun violence. Yet, there are at least three gaps that exist in the existing literature surrounding racial and ethnic disparities in gun violence that limit our understanding of why these disparities take the racial, gender, and age-graded shape they do including: (1) problems associated with data on ethnicity; (2) the lack of data on non-fatal firearm injury; and (3) the lack of comprehensive data collections that are inclusive of the myriad of factors associated with disparities in gun violence.

The first research gap concerns the lack of consistent, longitudinal data among Hispanics, an issue which has plagued criminology more generally [see (28)]. Existing data collections have not consistently collected information on Hispanics, sometimes lumping this group with White people, other times separating them out entirely. Without a consistent data collection system that appropriately categorizes Hispanics, we will continue to have a lack of understanding about gun violence trends among that group.

Second, existing data sources that have tracked trends in gun violence have tended to focus primarily on firearm homicides, leaving a lack of consistent, longitudinal data on non-fatal gun violence. Fortunately, in September 2024, the (now defunct) White House Office of Gun Violence Prevention announced two improvements to the federal data infrastructure surrounding gun violence (29). First, the FBI-based National Incident Based Reporting System (NIBRS) data collection established a new injury code to reflect a gunshot wound in the NIBRS victim segment and a mechanism for law enforcement to submit additional information on how the firearm was used in crime. A second effort led by the Centers for Disease Control (CDC) is designed to provide quick access to gun death and injury data faster and, perhaps even more importantly, to have that available at a more local level in a dashboard format. These are two small, federal steps toward the provision of more informative and timely gun violence data and it is up to researchers to undertake the research that will help illuminate the trends and patterns that these data evince.

A third research gap centers on conducting rigorous analyses into the causes and correlates associated with the racial and ethnic disparities surrounding gun violence. These disparities are not recent, although they were exacerbated during the COVID-19 pandemic, a time period whose closures moved dispute dynamics online while grief accumulated, which raised the question as to whether the risk factors that are commonly associated with (gun) violence were simply heightened among Black people. Other criminological theories, such as routine activities theory may also be useful on this topic. Roman (12) has argued that the pandemic brought about school and business closures that disproportionately impacted young people, especially those who lived in places with a history of trauma and violence (which tend to favor disadvantaged Black people) thereby leaving juveniles with serious disputes in close proximity to each other. It is possible that adding Roman's suggestions alongside the online migration of conflict and the acute amplification of toxic stress, grief, and legal cynicism during a period of highly publicized police-involved deaths may also have contributed to the surge in Black firearm homicides noted by Piquero and Roman. And while there has been some reversal of the violence spike during the pandemic, better analysis is needed at both the community- and especially individual-levels to assess how the reopening of social institutions coupled with the large federal investments in communities and violence prevention may have affected these patterns and individual decisions to use firearms to settle disputes.

## Potential future developments

Aside from addressing the research gaps highlighted in the previous section, it has historically been the case that attention paid to demographic disparities in gun violence has tended to focus on community and economic disadvantage that creates the conditions conducive to gun violence. In fact, Lee et al.'s (30) recent work also shows that racialized economic segregation was associated with higher perceived community violence, which was then associated with firearm carrying, a finding which resonates with Anderson's street code thesis as well as the more general work surrounding culture and legal cynicism (31) especially in neighborhoods marked by concentrated disadvantage (32).

Although this is certainly an important consideration, equally important is the recognition that gun violence diffuses among people and across places through social relationships (33), a type of social contagion if you will that has been shown to be more pronounced among minority males (34). Continued work in this area regarding just how this contagion works at the local level and how this contagion, in turn, shapes individual decision-making is an appropriate step.

An additional area of future development that may help provide unique insights into gun violence would take a page from the playbook of a developmental perspective, which emphasizes the interplay of biological, psychological, social, and ecological factors across an individual's lifespan (35). Within the field of developmental and life-course criminology, it has been well-accepted that involvement in antisocial, criminal, and especially violent activity cannot be understood without full appreciation for the interplay of the ebbs and flows of causes and correlates of offending throughout the life course, from birth through adulthood (36, 37). Insights from this developmental perspective have shown, for example, that the risk and protective factors (e.g., delinquent peer networks, family structure, self-control, prosocial relationships, educational attainment) associated with crime and violence may be age-graded such that their relationship to offending may be stronger or weaker at different periods of the life course (e.g., childhood, adolescence, early adulthood, mid-adulthood, and late-adulthood). Particularly instructive here is the work on adverse childhood experiences [ACEs; (38)], which have been found to be both a type of adverse childhood experience (39) as well as a correlate of exposure to firearms (40). As well, an increasing amount of research attention has noted the importance of a biopsychosocial perspective that has shown the complex interplay of these factors as they pertain to adolescent risk-taking where reward-seeking and capacities for self-control develop differently, the former much sooner than the latter (41). Increasingly, researchers have also started to examine the dynamic interplay between early (social) adversity within distressed neighborhoods—especially those that are exceedingly violent. Combining multiple strands of data sources, including data from the multi-cohort longitudinal Project on Human Development (PHDCN) study, Manduca and Sampson (42) found that exposure to neighborhood violence, incarceration, and lead were related to adult incarceration risks among poor Black youth among other adverse outcomes. In another study, Piquero et al. (43) used data from the Baltimore site of the National Collaborative Perinatal Project to test the argument that individual (e.g., low birth weight) and familial (e.g., whether mother received public assistance at birth of child, age at mother at childhood) risks are exacerbated in the most disadvantaged communities (e.g., poverty, public assistance, etc.)—especially those that are overrepresented by Black persons. Their race-specific analysis revealed that individual and familial risk interacted to predict the most severe forms of offending—but only for Blacks—and the effect was magnified in disadvantaged neighborhoods among Blacks. With advances in data collection, researchers have increasingly started to focus on a variety of related factors such as'toxic stress', allostatic load, the effects of chronic inflammation on the brain, and neighborhood disadvantage and across race/ethnicity [see e.g., (45–47)]. Of course, bringing evidence to bear on this point requires longitudinal data which has the promise of helping researchers assess the cumulative effects and timing of racism experiences on firearm violence. Longitudinal studies can also highlight how experiences of racism and its downstream consequences shape firearm violence differently across life stages across the life span. This type of data collection and ensuing analysis is important because much of the work highlighted throughout this review—as well as the integrated cascading perspective advanced herein—has not been established at the individual level of analysis over time.

Equally important, but much less studied, is how gun violence – particularly its concentration among racial and ethnic minorities (and mainly in distressed inner-city communities) – also influences individual health which can, in turn, further exacerbate the already high racial health disparities. Recently, Semenza et al. (48) used survey data from a national sample to examine this issue, finding that minority (Black and Hispanic) adults were exposed to higher gun violence exposure and community disadvantage than White people and that this gun violence exposure was associated with poorer self-rated and chronic health (p. 1). In short, continued attention to the long-term consequences of youth exposure to firearm injury and death is vitally important to help inform prevention efforts (49).

Lastly, it is important that what is gained from analysis of data on racial and ethnic disparities in gun violence helps to inform policy responses that seek to attend to the community, familial, situational, and individual levels of analysis as it is likely that an intermix of risk factors at all four levels helps to create the right conditions for gun violence. Attention is urgently needed at the individual-level of analysis because people still make a decision to pick up a gun, carry a gun, and then discharge the firearm upon another person or persons. This is where we need to understand why people make the decisions to use a firearm in a specific context. From there, consideration can then be given to arriving at equitable policy solutions: (1) structural such as systemic change and reform, including constitutionally just policing and investment in preventive measures such as health, education, and employment opportunities (50, 51); (2) community-based efforts such as community violence interventions, hospital-based interventions, and grassroots initiatives (52, 53); (3) leveraging the SAFE Lab's community-informed computation approach [e.g., (54, 55)], for example, by exploring social-media informed violence interruption in which outreach workers monitor online escalation to interrupt retaliation before it moves offline; and (4) individual prevention efforts such as self-control modification and early family parent training programs (56, 57), the Becoming a Man Initiative (58) and Stop Now and Plan [SNAP; (59)] that have been found to promote self-control and better decision-making and, in turn, lower delinquent and criminal activity as well as gun violence Importantly, the individual-level programs noted here are interventions focused on the social-cognitive threat-appraisal and impulse-regulation processes implicated in gun violence while the social-media-informed violence interruption extends community violence intervention to where disputes now escalate—online. Ideally as well, these programs would be paired with (the more difficult) structural change - reducing the toxic stress and legal-cynicism inputs - rather than deployed alone.

All of this is to suggest that racial and ethnic disparities in gun violence are likely to involve a complex mixture of historical, structural, community, situational, and individual factors which implies, of course, that prevention and intervention approaches must pay close attention to all of these levels of analysis and do so within a developmental perspective that considers their interplay throughout the life course. Undoubtedly, transdisciplinary theoretical models, such as the one advanced by the National Institute on Minority Health and Health Disparities research framework, are a prime candidate to help unpack the reasons underlying these racial disparities and why such disparities are highly stable (60).

## Footnotes

1 Lee et al. (62) recently conducted a scoping review to synthesize the current evidence on the relationship between racism and firearm violence. The results of their review revealed that residential racial segregation, historical redlining, racialized economic segregation, racial inequalities in socioeconomic outcomes, and other novel measures of racism (e.g., historical enslavement rate, the killing of George Floyd) were identified as risk factors for firearm violence (p. 314). In particular, the most common and consistently identified risk factor, residential racial segregation, was noted as having indirect effects on firearm violence as it operates by limiting “racially minoritized communities from accessing key economic and societal resources but also foster deleterious social conditions, such as community violence, which increases the likelihood of firearm violence” [Lee et al. (62), p. 21]. While their scoping review was at a larger level of analysis, the index of concentration extremes helps to take the more structural disparity phenomenon to a place-based level by calculating how residents are concentrated into groups at the extremes of deprivation and privilege (63).

2 To be sure, Carlson's work relies on interviews and interpretive and should be considered alongside the epidemiological research.

3 It is worth noting here as well that cultural adaptations and legal cynicism could act as a potential mediating mechanisms between structural racism and privatized armed protection. For example, Burrell et al. (61) developed a model outlining the causal structures that drive dynamics of community violence escalation over time in a context of historical racism among Black urban males to help illuminate the violence feedback loops that occur in certain communities.
